# Multivariate Analysis among Marker Compounds, Environmental Factors, and Fruit Quality of *Schisandra chinensis* at Different Locations in South Korea

**DOI:** 10.3390/plants12223877

**Published:** 2023-11-16

**Authors:** Dong Hwan Lee, Young-Ki Kim, Yonghwan Son, Gwang Hun Park, Hae-Yun Kwon, Youngki Park, Eung-Jun Park, Sun-Young Lee, Hyun-Jun Kim

**Affiliations:** 1 Forest Medicinal Resources Research Center, National Institute of Forest Science, Yeongju-si 36040, Republic of Korea; leedh0419@korea.kr (D.H.L.); treeace@korea.kr (Y.-K.K.); thsdydghks@korea.kr (Y.S.); ppkh0230@korea.kr (G.H.P.); kwonhy05@korea.kr (H.-Y.K.); nararawood@korea.kr (S.-Y.L.); 2 Department of Forest Bioresources, National Institute of Forest Science, Suwon 16631, Republic of Korea; woodpark@korea.kr (Y.P.); pahkej@korea.kr (E.-J.P.)

**Keywords:** correlation analysis, growth characteristics, magnolia berry, marker compounds, method validation, UPLC-UV

## Abstract

This study aimed to investigate the correlation among the contents of marker compounds, growth characteristics, and environmental factors of *Schisandra chinensis* fruits across South Korea. The fruits were collected from 36 cultivation sites in 28 regions across the country. We investigated nine growth characteristics, twelve soil physicochemical properties, eight meteorological data, and three marker compounds in this study. We optimized and validated an optimized method for quantifying marker compounds using UPLC and performed correlation analysis among the contents of marker compounds, growth characteristics, and environmental factors. The UPLC-UV method for analyzing marker compounds was validated by measuring linearity, LOD, LOQ, precision, and accuracy. The marker compounds were negatively correlated with the fruit size and sugar contents, and growth characteristics were negatively correlated with some physicochemical properties of the soil. The results of this study can be used as basic data for the standard cultural practices and quality control of *S. chinensis* fruits.

## 1. Introduction

Natural products are currently one of the most numerous and highly valued ingredients in the pharmaceutical and cosmetic industries [1]. There are numerous natural products in plants, and these natural products are known to have various physiological active functions such as antioxidants, anticancer, and anti-inflammatory [2,3]. Natural products offer special functions compared to conventional synthetic molecules, which give both benefits and challenges to the drug discovery process [4]. The medicinal applications of natural products to improve human health were first recorded in 2900–2600 BC, and several documents have been discovered since then [4,5]. About 80% of all medicines were derived from plant sources by the early 1900s [5]. Since then, many studies on natural products have been conducted, and to date, about 60% of approved small-molecule drugs are related to natural products, and 69% of antibacterial agents are derived from natural products [6]. Traditional medicines using natural products have also been practiced in Asia. Five-flavor fruit is also a plant that has been used as a traditional medicinal material in Korea and China [7].

*Schisandra chinensis* (Turcz.) Baill. is called magnolia berry, five-flavor fruit, omiza (in the Republic of Korea), wu-wei-zi (in China), gomishi (in Japan), and Limonnik (in Russia) [8]. It belongs to the Schisandraceae family, which is distributed in Korea, Northeast China, and the Russian Far East [8]. In East Asia, Schisandraceae has been used in traditional treatments for ailments such as chronic coughs, dyspnea, diarrhea, and enuresis [7,9,10]. Additionally, various physiological functions, such as antibacterial, alcohol detoxification activity, antiaging, antitumor, immunomodulatory, anti-HIV, protection from cardiovascular disease, and the regulation of the CNS have been reported in relation to the use of *S. chinensis* [8,11,12,13,14,15]. These activities are known to be caused by the metabolites present in the plants [16]. So far, research is being conducted on the lignan of *S. chinensis* fruits, such as gomisin, schisandrin, deoxyschisandrin, schisandrol, etc. [17]. Lignans are a class of secondary metabolites widely distributed in the plant kingdom and found in species belonging to more than 70 families [16]. Lignans in plants have various bioactive activities such as anticancer, anti-inflammatory, antimicrobial, antioxidant, and immunosuppressive activities [16,18]. It has been reported that lignans from *S. chinensis* exert cardiovascular protective activities by controlling multiple signaling pathways involved in various biological processes such as vascular contractility, fibrosis, apoptosis, oxidative stress, and inflammation [15]; therefore, the lignans of *S. chinensis* are very important from a pharmacological perspective. In Korea, three lignans (schisandrin, gomisin A, and gomisin N) have been noted as being marker compounds of *S. chinensis* fruit in the Korean Pharmacopoeia [19].

Generally, marker compounds are affected by plant growth, which is determined by soil physicochemical properties, climate, soil microorganisms, and the like [20,21]. However, research on environmental factors to increase the lignan content of *S. chinensis* is lacking. Therefore, it is necessary to study the effect of plant growth on the contents of compounds and the effect of environmental factors on plant growth for quality control of S. chinensis fruits used as raw materials in the pharmaceutical industry. However, to our knowledge, few such studies have been reported. In this study, we focused on the correlation among the contents of lignans, growth characteristics, and environmental factors of *S. chinensis* fruits and cultivation sites.

## 2. Results

### 2.1. Soil Characteristics and Meteorological Factors

The physicochemical properties of 12 soils were analyzed (Table 1). As a result of soil texture analysis, it was confirmed that those were loam, sandy loam, clay loam, and sandy clay loam. For soil pH, the highest value was pH 7.42 ± 0.25 at site 18, and the lowest value was pH 4.08 ± 0.04 at site 35. Sixteen sites belong within the soil acidity range (pH 5.5 to 6.5) suitable for tree growth, and it was found that the values of electrical conductivity were suitable (<1.0 dS/m) at all sites [1]. The content of organic matter (OM), total nitrogen (TN), and available phosphate (AP) were significantly higher at site 29 than at other sites. In the case of cation exchange capacity (CEC), the highest was confirmed at site 8, at 36.12 ± 3.78 cmol^+^/kg, and the lowest was confirmed at site 2, at 7.61 ± 3.00 cmol^+^/kg. In this study, 25 sites belonged to the suitable range (12‒20 cmol^+^/kg) for tree growth. The value of base saturation (BS) was the highest at site 36 at 130.71 ± 14.65% and the lowest at site 35 at 11.16 ± 2.77%.

The meteorological data were collected in relation to seven factors (Table S1). The annual average temperature (AAT) was in the range of 9.6‒13.9 °C. The annual average maximum temperature (AAMT) was in the range of 15.0‒19.7 °C, and the annual average minimum temperature (AAmT) was in the range of 4.9‒9.7 °C. The highest value in annual maximum temperature (AMT) was 36.4 °C at site 14 and site 18, and the lowest value in annual minimum temperature (AmT) was −20.3 °C at site 5. The values of the annual total precipitation (TP) were highest at site 1 at 2136.8 mm and lowest at site 35 at 1128.9 mm. The values of the annual sum of sunshine hours (SSH) were highest at site 33 at 2444.2 h and lowest at site 21 at 1901.8 h.

### 2.2. Growth Characteristics

The growth characteristics data of *S. chinensis* fruits are shown in Table 2. The greatest number of fruits per fruit bunch was evident in the Boeun area (site 34), at about 34, and the least was in the Inje (site 2) and Gapyeong areas (site 5), at about 21. Roughly, the Boeun area (76.07 ± 5.17 mm, 25.13 ± 1.39 mm, 22.33 ± 2.58 g) yielded the largest size in terms of fruit bunch, concerning the length (LB), width (WB), and fresh weight of fruit bunch (FWB), and the smallest fruit bunch was in the Namwon area (site 25, 44.48 ± 3.48 mm, 19.60 ± 1.48 mm, 8.62 ± 1.24 g). The largest fruit size (LF, WF, FWF, and FW30F) was confirmed at the Pyeongchang area (site 4, 12.48 ± 0.09 mm, 10.64 ± 0.19 mm, 0.91 ± 0.02 g, 28.39 ± 0.51 g), and the smallest was at the Namwon area (site 25, 9.52 ± 0.74 mm, 8.27 ± 0.55 mm, 0.49 ± 0.06 g, 15.39 ± 1.38 g). The sugar content (SG) of fruit was the highest in the Cheongju area (site 35) at 12.23 ± 0.77 Brix°, and the Sancheong area (site 8) was the lowest at 4.74 ± 0.17 Brix°.

### 2.3. Validtaion and Quantitative Analysis of Marker Compounds

The established UPLC methods were also validated to ensure their suitability for the quantitative analysis, and these are shown in Table 3, Table 4 and Table 5. The linearity showed good correlation coefficients exceeding 0.9999 for all of the marker compounds. The limit of detection (LOD) and quantification (LOQ) values of three marker compounds (schisandrin, gomisin A, and gomisin N) were 0.003 and 0.01 µg/mL, 0.02 and 0.07 µg/mL, and 0.01 and 0.04 µg/mL, respectively (Table 3). Intra-day and inter-day variations were calculated by measuring the amounts of the three marker compounds three times at three different concentration levels. Relative standard deviation (RSD) values were calculated as a percentage of the standard deviation divided by the mean and ranged from 0.02% to 0.52% (intra-day) and 0.23 to 2.50% (inter-day), which are within an acceptable range (Table 4). The recovery of the marker compounds was calculated to 98.40‒102.68% and an RSD of <3.0% for accuracy (Table 5). These results validate the analytical method optimized for the analysis of the contents of marker compounds in *S. chinensis* fruits using UPLC.

**Figure 1:**
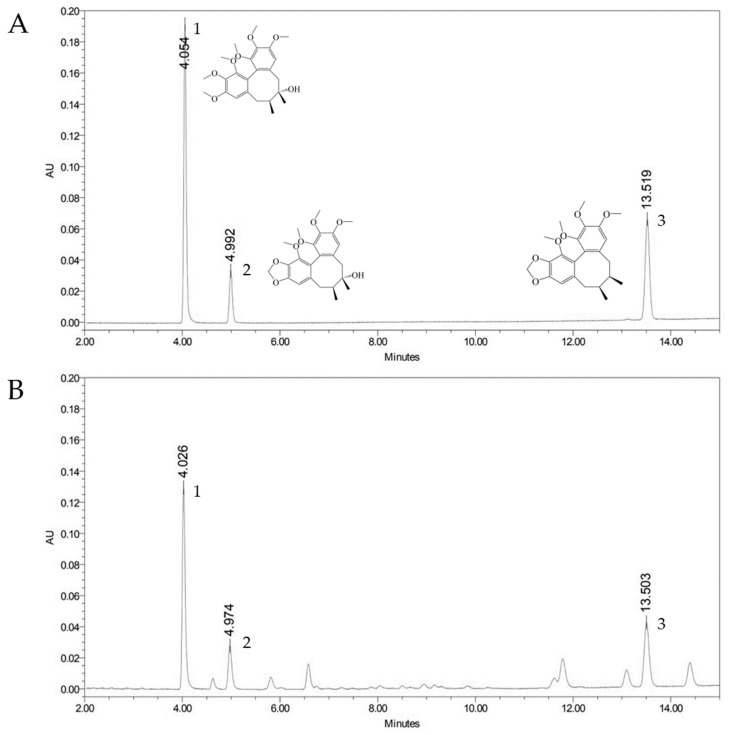
UPLC chromatogram of marker compounds of the standard mixture (**A**) and *Schisandra chinensis* fruit sample (**B**). 1, Schisandrin; 2, Gomisin A; 3, Gomisin N.

The 108 samples of *S. chinensis* fruits were analyzed using the UPLC method. Marker compounds were identified by comparing the UV spectra chromatograms and retention times of the peaks with standard solutions. The three marker compounds (schisandrin, gomisin A, and gomisin N) were detected at a retention time of 4.054, 4.992, and 13.519 min, respectively (Figure 1). The quantitative results are shown in Table 6. The samples contained schisandrin from 0.41 ± 0.00 to 0.79 ± 0.03%, gomisin A from 0.08 ± 0.01 to 0.49 ± 0.02%, gomisin N from 0.23 ± 0.01 to 0.55 ± 0.02%. The highest content of schisandrin was confirmed at site 14, gomisin A at site 8, and gomisin N at site 7. The highest total content of marker compounds was confirmed to be 1.75 ± 0.01% at site 8.

### 2.4. Correlation Analysis and Network Model

**Figure 2:**
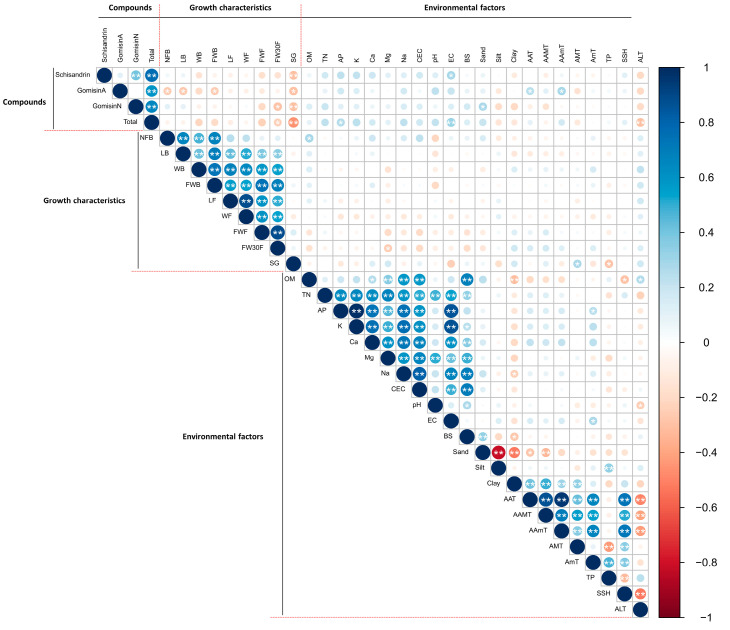
Correlation analysis of marker compounds, growth characteristics, and environmental factors. * *p* < 0.05, ** *p* < 0.01.

**Figure 3:**
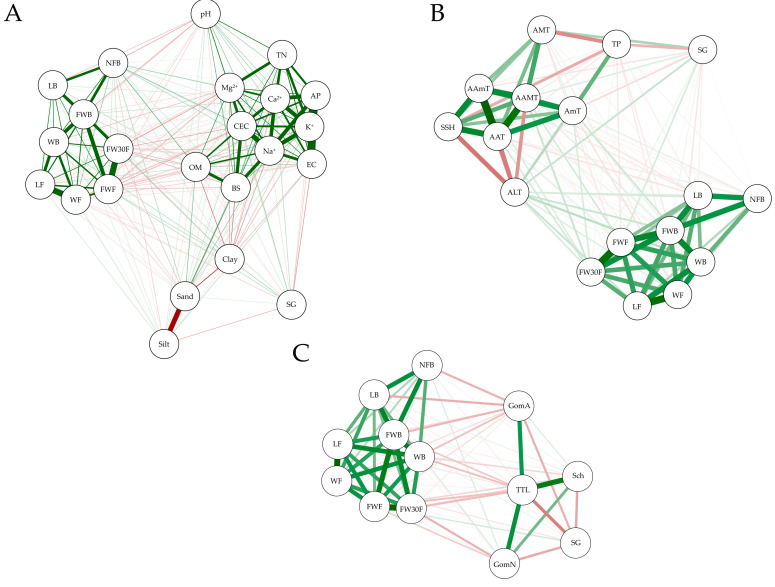
Network model for relationship between growth characteristics and environmental factors ((**A**): soil physicochemical properties; (**B**): meteorological factors) and contents of marker compounds (**C**); green lines, positive correlations; red lines, negative correlations; Sch, Schisandrin; GomA, Gomisin A; GomN, Gomisin N; TTL, Total.

Pearson correlation analysis between growth characteristics of *S. chinensis* fruits and environmental data (soil physicochemical and meteorological factors) was carried out (Tables S2 and S3, Figure 2). The number of fruits per fruit bunch (NFB) showed a positive correlation with OM (0.287, *p* < 0.003), CEC (0.226, *p* < 0.019), and sand (0.210, *p* < 0.029) and a negative correlation with soil pH (−0.213, *p* < 0.027). A fresh weight of fruit bunch (FWB) also has a negative correlation with soil pH (−0.208, *p* < 0.031). A fresh weight of fruit (FWF) and fresh weight of 30 fruits (FW30F) have a negative correlation with Mg^+^ (−0.197, *p* < 0.041; −0.258, *p* < 0.007) and CEC (−0.194, *p* < 0.045; −0.202, *p* < 0.036). Additionally, SG has a negative correlation with EC (−0.241, *p* < 0.012) and TP (−0.283, *p* < 0.003), whereas a positive correlation with AMT (0.272, *p* < 0.004). The correlation between the growth characteristics of *S. chinensis* fruit and environmental factors was visualized through a network model (Figure 3). Nodes represent each factor, and edges represent unique associations between factors. The network model (Figure 3A) shows that SG has only a weak correlation with EC, and there appears to be no particular correlation between the growth characteristics of the fruit and the physicochemical properties of the soil. The relationship between growth characteristics and weather factors showed that SG had a positive correlation with AMT and a negative correlation with TP (Figure 3B).

Correlation analysis between the content of marker compounds and growth characteristics of *S. chinensis* fruit was also performed (Table S4). The contents of gomisin A have a negative correlation with NFB (−0.257, *p* < 0.007), LB (−0.266, *p* < 0.005), WB (−0.193, *p* < 0.045), and FWB (−0.254, *p* < 0.008). Additionally, gomisin N has a negative correlation with FWF (−0.205, *p* < 0.033) and FW30F (−0.291, *p* < 0.002). A SG was negatively correlated with three marker compounds (schisandrin, −0.307, *p* < 0.001; gomisin A, −0.293, *p* < 0.002; gomisin N, 0.308, *p* < 0.001), and the sum of them (total, −0.450, *p* < 0.000). In the network model (Figure 3C) between the content of marker compounds and growth characteristics of *S. chinensis* fruit, gomisin A, gomisin N, and total contents appeared to be correlated with growth characteristics, and SG was shown to be correlated with all marker compounds. In summary, gomisin A had a negative correlation with NFB, LB, and FWB, and gomisin N had a negative correlation with FW30F and SG, and the total content was expressed as correlating with SG, FWB, WB, FWF, and FW30F, the results were similar to the Pearson’s correlation coefficient analysis.

## 3. Discussion

*S. chinensis* is an economically important species rich in nutrients and is widely used in herbal medicine and functional foods. Various active substances, including lignans, are related to the quality and economic value of *S. chinensis*. In this study, we tried to confirm the effect of the environment in the cultivation site on the growth of *S. chinensis* fruits and see what changes there are in the content of the marker compounds as a result.

The physicochemical properties of the soil (Table 1) at the *S. chinensis* cultivation site were compared with the suitability for plant growth [22]. The pH values of 16 cultivation sites were within the appropriate range (pH 5.5‒6.5). The EC (<1.0) was confirmed to be suitable for all sites. OM (≥3.0) and TN (≥0.25) were at good levels at 27 and 17 sites, respectively. AP (≥100) was mostly suitable, except for two cultivation sites. In the case of exchangeable cations, Ca^2+^, Mg^2+^, and Na^+^, excluding K^+^, were not suitable for the appropriate range in most cultivation sites. On the other hand, CEC was confirmed to be within the appropriate range (12.0‒20.0 cmol^+^/kg) for plant growth in about two-thirds of the investigated sites. BS was in the range 11.16‒130.71%. BS had a positive correlation with soil pH (0.684, *p* < 0.000, Table S5, Figure 2). Generally, when the pH of the soil decreases, the concentration of exchangeable aluminum increases, which is known as a toxic substance that inhibits root development and plant growth by interfering with the absorption and movement of nutrients [23,24,25]. In addition, high soil OM improves nitrogen absorption in plants through nitrogen mineralization, increasing crop productivity [26,27]. Meteorological data were also collected (Table S1). The AAT ranged from 9.6 °C to 13.9 °C. The highest AAMT was 19.7 °C, and the lowest AAmT was 4.9 °C. The TP was also confirmed to vary from 1128.9 mm to 2136.8 mm. Additionally, SSH ranged from 1901.8 h to 2444.2 h. *S. chinensis* is known to grow well in wet sandy loam with no strong sunlight, well-drained, ventilated, and corrosive [28], but it has been confirmed that it sometimes grows in other environments. Environmental factors such as altitude, temperature, precipitation, soil acidity, soil nutrients, drainage, etc., act in very complex ways, and these heterogeneities have an impact on plant growth and secondary metabolites [29,30]. Although research has been conducted on how plant growth is changed by environments, more research is needed on the effects on the secondary metabolites. Jan et al. (2021) reported in a review paper the role of secondary metabolites in responding to biotic and abiotic responses through the regulation of transcription factors and genes involved in environmental stress tolerance [31].

The growth characteristics of *S. chinensis* fruit varied significantly among the different cultivation sites. The fruit bunches were confirmed to be the largest in the Boeun area (site 34), and the fruits were found to be the largest in the Pyeongchang area (site 4). As a result of the correlation between growth characteristics, it was confirmed that there was no significant effect between fruit size and SG (Table S6). There are various reasons affecting fruit quality, such as soil physicochemical properties, soil microbiome, genetic factors, etc [32,33,34,35]. These various complex factors interact simultaneously while plants grow, making it difficult to know which factor has the greatest impact on plant growth. 

Analytical methods for the quantitation of marker compounds of *S. chinensis* fruits were validated in the present study. Validation results for linearity, LOD, LOQ, precision, and accuracy confirmed that the analytical method yielded reproducible and reliable results. Using a validated method, all marker compounds were detected within 14 min (Figure 1). Overall, it was confirmed that schisandrin was the most plentiful marker compound. Although there were differences in the ratio of marker compounds in the 36 cultivation sites, the sum of the three marker compounds was found to be 0.82~1.76%, exceeding the Korean Pharmacopoeia standard of 0.7% [19]. These results were similar to the previously reported 0.8–1.2% of 26 cultivation sites in Korea [36]. 

As a result of the correlation analysis of *S. chinensis* fruit marker compounds and growth characteristics, gomisin A has a negative correlation with fruit bunch growth, gomisin N has a negative correlation with fruit growth (NFB, LB, WB, FWB, FWF, and FW30F), and SG has a negative correlation with all marker compounds. These results were comparable to previous studies that reported a negative correlation between plant growth and the content of secondary metabolites. Ma et al. (2023) reported that goji berry contains the highest number of flavonoids when it is a green fruit, but as it matures, the content of flavonoids decreases [37]. Phan et al. (2022) reported that total soluble solid and titratable acidity increased depending on the ripening stage of Kakadu plum fruits, but the content of phenolic compounds, including tannin, tended to decrease [38]. In addition, our previous study [39] showed that the content of active compounds in the fruit of *Cudrania tricspidata* decreases as the size of the fruit increases. The correlation between fruit growth and environmental factors was also analyzed. The results showed that OM influenced NFB, Mg and CEC affected fresh weight, and factors such as AMT and TP impacted SG, respectively. In previously reported studies, the electrical conductivity of non-substituted soil was less than 2.0 dS/m based on salt soil, and the replaceable sodium ratio was less than 15% [40,41]. In this study, the highest electrical conductivity collected at cultivation sites was 0.75 ds/m and 2.19% in the highest exchangeable sodium percentage. Potassium is an essential nutrient element for plants and has an impact on growth and development. In soil, magnesium exists as a cation and competes with other ions such as K^+^, Ca^2+^, Mn^2+^, and NH^4+^ root uptake [42], so if the uptake of magnesium exceeds that of potassium, plant growth may be hindered. Zhang et al. (2018) reported that the temperatures (average, minimum, and maximum) from April to October affect the most positively on the quality of the Fuji apple, followed by annual average temperature and sunlight, daily temperature difference, and total precipitation [32]. Yuri et al. (2023) reported that when ‘Forelle’ pears were exposed to solar radiation energy, the total phenol content and antioxidant activity were higher than those of the control, and there was no difference in flesh firmness or total soluble solid content [43]. These studies confirm that the best growing conditions of plants and the environmental conditions with the highest content of secondary metabolites may not be the same. Therefore, it is possible to proceed with cultivation by changing the cultivation conditions of edible or medicinal plants as needed.

## 4. Materials and Methods

### 4.1. Sampling of Plant Material and Chemicals

**Figure 4:**
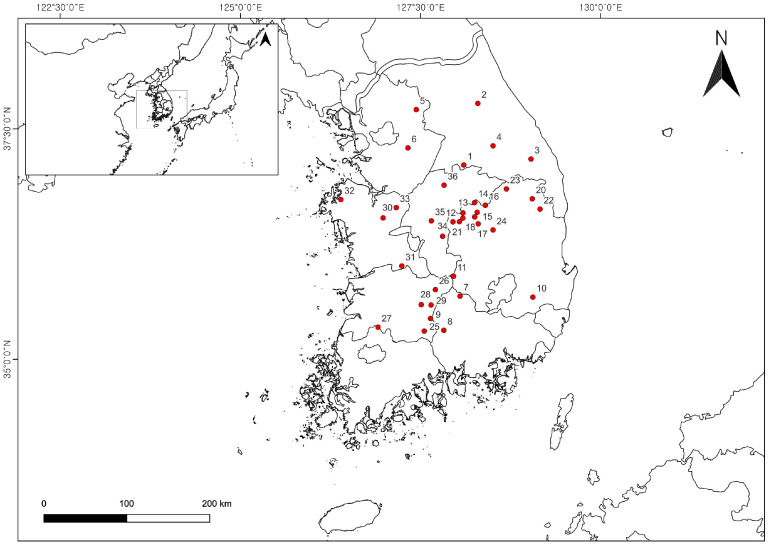
Geographical information for the cultivated fruit samples of *Schisandra chinensis* from the various provinces.

In September 2021, 108 samples of *S. chinensis* (specimen number: FMRC-B2109001~B2109108) fruits and soils from 36 cultivation sites were collected in 28 regions across South Korea (Table S7, Figure 4). Voucher specimens in this study have been deposited at the Forest Medicinal Resources Research Center herbarium (FMRC), National Institute of Forest Science in Korea. The moisture content of *S. chinensis* fruits was 81.60 ± 2.29%. Ten growth characteristics of *S. chinensis* fruit such as number of fruits per fruit bunch (NFB), length of fruit bunch (LB), width of fruit bunch (WB), fresh weight of fruit bunch (FWB), length of fruit (LF), width of fruit (WF), fresh weight of fruit (FWF), fresh weight of 30 fruits (FW30F), and sugar content (SG) were measured using digital calipers (500-182-30, Mitutoyo Co., Kawasaki, Japan), an electronic scale (HS3200S, HANSUNG instrument Co., Gwangmyeong, Republic of Korea), and a refractometer (PR-101α, ATAGO Co., Ltd., Tokyo, Japan). Standards of schisandrin and gomisin A were purchased from Sigma-Aldrich (St. Louis, MO, USA). Gomisin N was purchased from ChemFaces (Wuhan, Hubei, China). HPLC-grade acetonitrile, methanol, and distilled water were purchased from J.T. Baker (Avantor, Inc., Radnor, PA, USA) and used without purification.

### 4.2. Sample and Standard Preparation

The collected samples were washed and then lyophilized. The powder was pulverized with a grinder (KSP-35, Korea Medi Co., Ltd., Daegu, Republic of Korea) and stored at −18 °C before being used as an analysis sample.

Dried powder (500 mg) was extracted via ultrasonication in 10 mL of 100% MeOH in a 15 mL tube for one hour at room temperature. An ultrasonic bath (JAC-5020, KODO, Hwaseong, Republic of Korea) was set to 350 W output power and 40 kHz frequency. The extracts were centrifuged for 15 min at 1763× *g*, and the supernatant was separated with filter paper (ADVENTEC^®^ No. 2, Toyo Roshi Kaisha, Ltd., Tokyo, Japan). The samples were filtered with a 0.2 µm syringe filter (PTFE, 6784-1302, Whatman Co., Maidstone, UK) before UPLC analysis. Standard (schisandrin, gomisin A, and gomisin N) stock solutions for analysis were prepared by diluting the stock solutions in methanol to obtain concentration ranges of 6.25–400 µg/mL for all compounds. 

### 4.3. UPLC Analysis

Waters Alliance UPLC^®^ (Waters Co., Milford, MA, USA) with a PDA detector was used for the simultaneous analysis if three marker compounds in *S. chinensis* fruit. The analytical conditions for the three compounds were as follows: quantitative analysis was carried out using an ACQUITY UPLC system equipped with BEH C18 column (2.1 × 100 mm, 1.7 μm, 130 Å, Waters Co., Milford, MA, USA) with a column oven at 30 °C. The mobile phase used a binary eluent of 0.1% formic acid in water (A) and 0.1% formic acid in ACN (B) with gradient conditions as follows: initial: 3 min, 45% B; 3–5 min, 52% B; 5–6 min, 53% B; 6–8 min, 58% B; 8–10 min, 64% B; 10–15 min, 64% B; 15–15.1 min, 76% B, 15.1–17 min, 100% B; injection volume of 1 μL, flow rate of 0.3 mL/min and detection wavelength of 254 nm. All the chromatographic analyses were performed using the software for instrument control and data acquisition Empower 3 (Waters Co., Milford, MA, USA). Each sample was analyzed in triplicate and expressed as a mean value.

The method was validated for linearity, limit of detection (LOD), limit of quantitation (LOQ), precision (inter- and intra-day), and accuracy following the International Conference on Harmonization (ICH) guidelines [44]. Standard calibration curves were constructed with seven different concentrations from the following concentration ranges: 6.25–400 μg/mL for the three compounds (schisandrin, gomisin A, and gomisin N). LOD and LOQ under the present chromatographic conditions were determined at a signal-to-noise 3.3 and 10, respectively. Precision (%) was assessed for repeatability, intra-day (within one day), and inter-day (successive three days) and reported as the relative standard deviation (RSD, %). Accuracy (%) was assessed using a tested recovery assay by analyzing the peak areas of sample extracts with standard stock solution added and those without stock solution added. Each sample was analyzed in triplicate at three different concentrations and expressed as a mean value.

### 4.4. Soil Analysis and Meteorological Data

Soils were collected at a depth within 20 cm after removing surface soils at cultivation sites. Collected soils were dried at room temperature and stored after passing through a 2 mm standard sieve. Soils were classified according to United States Department of Agriculture (USDA) specifications into 12 classes of soil texture classification. The soil physicochemical properties analyses, such as soil pH, electrical conductivity (EC), organic matter (OM), total nitrogen (TN), available phosphate (AP), exchangeable cation (K^+^, Ca^2+^, Mg^2+^, and Na^+^), and cation exchange capacity (CEC) were performed according to the standard analysis manual of the Rural Development Administration (RDA) in Korea. Soil pH and EC were measured by adding 10 g of dried soil to 50 mL of distilled water and subsequently using a pH meter and an EC meter, respectively. The soil OM content was measured using the Tyurin method, and the TN content was measured via the Kjeldahl sulfuric acid distillation method. AP was measured using the molybdenum blue method using 1-amino-2-naphthol-4-sulfonic acid solution. After leaching dried soil sample in 1N NH_4_OAc (pH 7.0), the exchangeable cation was measured using an inductively coupled plasma optical emission spectrometry (ICP-OES) and the CEC of the exchanged ammonium was measured using the Kjeldahl distillation method. Each sample was analyzed in triplicates and expressed as a mean value. Base saturation (BS) was calculated as a percentage of exchangeable cations divided by CEC. Each sample was analyzed in triplicate and expressed as a mean value.

Data on weather conditions at the different cultivation sites were quoted from the Korea Meteorological Administration’s open portal (data.kma.co.kr, accessed on 1 January 2023). The collected data were annual average temperature (AAT), annual average maximum temperature (AAMT), annual average minimum temperature (AAmT), annual maximum temperature (AMT), annual minimum temperature (AmT), total precipitation (TP), and the sum of sunshine hours (SSH). 

### 4.5. Statistical Analysis

Statistical analysis was performed using SPSS software (Statistical Package for the Social Science, Version 26, IBM SPSS Statistics, Chicago, IL, USA), and data were expressed as mean ± standard error (S.E.). Statistical analyses of the results were performed at a 5% significance level. Multivariate analysis of variance (MANOVA) and Tukey’s multiple comparison tests were used to detect differences between the groups. The correlation among growth characteristics, marker compounds of *S. chinensis* of fruit, and the environmental factors of the cultivation site was confirmed through the use of Pearson’s correlation coefficient. Visualization of the correlation and network model was generated using the ggcorrplot2 and qgraph packages in R studio (Version 2023.06.1, Posit PBC, Boston, MA, USA).

## 5. Conclusions

In this study, we wanted to find out how the growth of *S. chinensis* fruit changes depending on the environment and how these changes affect the lignan content. We have investigated the growth characteristics and marker compound contents of *S. chinensis* fruit at 36 cultivation regions across South Korea. The simultaneous method of marker compounds of *S. chinensis* fruit was optimized based on the UPLC. The method was validated with linearity, LOD, LOQ, precision, and accuracy. The correlation among marker compounds, growth characteristics, and environmental factors was also analyzed. Gomisin A had a negative correlation with fruit bunch size, gomisin N had a with fruit size, and SG had with all compounds. Fruit size was negatively correlated with Mg and CEC, and SG was positively correlated with AMT and negatively correlated with EC and TP. There have been several existing studies that have directly confirmed the correlation between plant compounds and the environment, but there are very few studies that have investigated the correlation between marker compounds and growth, between growth and environmental factors. Growth characteristics are expressed in phenotypes and cannot be ignored because they indicate whether the environment is suitable for plant growth. The results of this study can be used to study standard cultivation practices and quality control of *S. chinensis* fruits used in the pharmaceutical industry. Further research on what biosynthetic pathways are stimulated by environmental factors to affect the growth and marker compound content of the plant is necessary in the near future.

## Figures and Tables

**Table 1:** Soil physicochemical properties of 36 different *Schisandra chinensis* cultivation sites.

Cultivation Sites (n = 3)	Soil Texture	pH [1:5]	EC	OM	TN	AP	Exchangeable Cation				CEC	BS
							K^+^	Ca^2+^	Mg^2+^	Na^+^		
			(dS/m)	(%)	(%)	(mg/kg)	(cmol^+^/kg)				(cmol^+^/kg)	(%)
1	loam	5.14 ± 0.08 ^ghijk^	0.14 ± 0.00 ^cd^	3.82 ± 0.28 ^cdef^	0.23 ± 0.03 ^defgh^	1985.12 ± 98.86 ^cdefg^	0.63 ± 0.11 ^cd^	3.88 ± 0.36 ^jkl^	0.65 ± 0.05 ^hij^	0.02 ± 0.00 ^c^	16.28 ± 0.56 ^efgh^	32.04 ± 3.43 ^hij^
2	sandy loam	6.33 ± 0.31 ^abcdefg^	0.06 ± 0.01 ^d^	0.78 ± 0.16 ^f^	0.06 ± 0.02 ^h^	91.03 ± 25.83 ^l^	0.19 ± 0.06 ^d^	5.20 ± 1.09 ^ghijkl^	0.79 ± 0.33 ^ghji^	0.17 ± 0.09 ^bc^	7.61 ± 3.00 ^i^	107.12 ± 29.95 ^abcdef^
3	sandy loam	7.02 ± 0.11 ^ab^	0.15 ± 0.01 ^cd^	4.68 ± 0.35 ^cdef^	0.28 ± 0.04 ^defgh^	1718.00 ± 64.89 ^defghij^	1.38 ± 0.07 ^cd^	9.97 ± 0.30 ^bcdefghijkl^	2.65 ± 0.19 ^cdefghi^	0.02 ± 0.00 ^c^	18.17 ± 0.74 ^defg^	77.29 ± 1.58 ^abcdefghi^
4	loam	5.12 ± 0.04 ^ghijk^	0.14 ± 0.01 ^cd^	5.12 ± 0.34 ^cdef^	0.29 ± 0.02 ^defgh^	2114.42 ± 91.25 ^cdef^	0.36 ± 0.03 ^cd^	3.67 ± 0.37 ^jkl^	0.53 ± 0.07 ^hij^	0.01 ± 0.00 ^c^	16.00 ± 1.19 ^efghi^	28.92 ± 3.24 ^ij^
5	loam	5.40 ± 0.67 ^efghij^	0.27 ± 0.20 ^bcd^	3.02 ± 0.85 ^cdef^	0.18 ± 0.07 ^efgh^	413.55 ± 126.95 ^hijkl^	0.94 ± 0.48 ^cd^	5.80 ± 2.14 ^fghijkl^	1.07 ± 0.38 ^fghij^	0.03 ± 0.00 ^c^	13.89 ± 0.85 ^efghi^	58.86 ± 21.15 ^cdefghij^
6	sandy loam	6.83 ± 0.07 ^abc^	0.25 ± 0.02 ^bcd^	12.90 ± 3.44 ^a^	0.68 ± 0.15 ^ab^	3519.01 ± 57.66 ^bc^	1.46 ± 0.21 ^bcd^	17.73 ± 2.83 ^bc^	5.49 ± 0.11 ^a^	0.03 ± 0.01 ^c^	26.12 ± 3.69 ^bcd^	96.18 ± 9.59 ^abcdefg^
7	loam	6.17 ± 0.16 ^bcdefgh^	0.14 ± 0.02 ^cd^	3.12 ± 0.30 ^cdef^	0.17 ± 0.03 ^efgh^	820.87 ± 90.95 ^efghijkl^	0.45 ± 0.10 ^cd^	8.79 ± 0.90 ^bcdefghijkl^	1.86 ± 0.41 ^cdefghij^	0.07 ± 0.02 ^c^	16.59 ± 0.98 ^efg^	68.37 ± 9.89 ^bcdefghij^
8	sandy loam	5.52 ± 0.10 ^defghi^	0.37 ± 0.08 ^bc^	11.78 ± 0.95 ^ab^	0.71 ± 0.07 ^ab^	1604.32 ± 228.73 ^efghijkl^	1.42 ± 0.09 ^bcd^	16.42 ± 3.93 ^bcd^	2.37 ± 0.59 ^cdefghij^	0.15 ± 0.02 ^bc^	36.12 ± 3.78 ^a^	54.97 ± 8.71 ^defghij^
9	loam	4.95 ± 0.20 ^hijk^	0.12 ± 0.01 ^cd^	5.49 ± 0.40 ^cdef^	0.26 ± 0.02 ^defgh^	76.04 ± 18.12 ^l^	0.55 ± 0.06 ^cd^	2.92 ± 1.30 ^kl^	0.20 ± 0.03 ^j^	0.31 ± 0.14 ^ab^	17.22 ± 0.59 ^efg^	22.61 ± 7.69 ^ij^
10	clay loam	5.94 ± 0.16 ^bcdefghi^	0.12 ± 0.01 ^cd^	3.01 ± 0.37 ^cdef^	0.15 ± 0.02 ^fgh^	430.56 ± 37.69 ^hijkl^	0.28 ± 0.02 ^cd^	8.57 ± 0.40 ^cdefghijkl^	2.98 ± 0.11 ^cdefg^	0.03 ± 0.01 ^c^	15.78 ± 0.23 ^efghi^	75.23 ± 2.51 ^abcdefghi^
11	sandy loam	5.89 ± 0.04 ^bcdefghi^	0.13 ± 0.02 ^cd^	4.65 ± 0.33 ^cdef^	0.25 ± 0.02 ^defgh^	1544.55 ± 39.91 ^efghijkl^	0.67 ± 0.06 ^cd^	5.23 ± 0.58 ^ghijkl^	2.29 ± 0.47 ^cdefghij^	0.01 ± 0.00 ^c^	16.88 ± 0.81 ^efg^	48.16 ± 4.25 ^fghij^
12	sandy loam	6.81 ± 0.06 ^abc^	0.15 ± 0.01 ^cd^	5.25 ± 0.06 ^cdef^	0.37 ± 0.02 ^defg^	1680.65 ± 55.97 ^defghijk^	0.97 ± 0.06 ^cd^	12.44 ± 0.35 ^bcdefghijk^	3.78 ± 0.28 ^abcd^	0.02 ± 0.00 ^c^	18.96 ± 0.95 ^def^	91.10 ± 2.94 ^abcdefgh^
13	sandy loam	5.39 ± 0.34 ^efghij^	0.34 ± 0.03 ^bcd^	11.06 ± 0.86 ^ab^	0.64 ± 0.07 ^abc^	2193.25 ± 268.63 ^bcde^	0.73 ± 0.16 ^cd^	13.69 ± 3.14 ^bcdefghi^	1.59 ± 0.36 ^defghij^	0.02 ± 0.01 ^c^	32.03 ± 3.32 ^ab^	48.86 ± 7.28 ^efghij^
14	sandy loam	6.25 ± 0.12 ^abcdefg^	0.10 ± 0.00 ^cd^	3.07 ± 0.12 ^cdef^	0.15 ± 0.01 ^fgh^	614.08 ± 52.79 ^fghijkl^	0.46 ± 0.05 ^cd^	5.94 ± 0.41 ^fghijkl^	0.80 ± 0.07 ^ghij^	0.09 ± 0.02 ^bc^	16.51 ± 1.12 ^efg^	44.38 ± 2.54 ^ghij^
15	sandy loam	6.27 ± 0.14 ^abcdefg^	0.27 ± 0.06 ^bcd^	4.93 ± 0.84 ^cdef^	0.30 ± 0.05 ^defgh^	2081.92 ± 404.85 ^cdef^	1.30 ± 0.27 ^cd^	13.71 ± 1.62 ^bcdefghi^	2.74 ± 0.61 ^cdefgh^	0.12 ± 0.03 ^bc^	19.03 ± 1.20 ^def^	93.21 ± 7.45 ^abcdefg^
16	sandy loam	5.76 ± 0.45 ^cdefghi^	0.75 ± 0.18 ^a^	7.88 ± 0.68 ^bc^	0.46 ± 0.02 ^bcd^	3712.87 ± 1149.72 ^b^	3.27 ± 1.51 ^a^	14.94 ± 3.76 ^bcdef^	3.87 ± 1.30 ^abc^	0.42 ± 0.15 ^a^	20.37 ± 1.15 ^def^	108.04 ± 27.30 ^abcdef^
17	sandy loam	6.94 ± 0.11 ^abc^	0.21 ± 0.03 ^cd^	5.98 ± 0.35 ^cde^	0.39 ± 0.02 ^cdef^	1817.50 ± 21.38 ^defghi^	1.72 ± 0.13 ^abcd^	15.58 ± 0.65 ^bcde^	5.36 ± 0.50 ^ab^	0.15 ± 0.04 ^bc^	20.13 ± 0.80 ^def^	114.06 ± 10.10 ^abcd^
18	sandy loam	7.42 ± 0.25 ^a^	0.24 ± 0.04 ^cd^	5.05 ± 0.35 ^cdef^	0.35 ± 0.03 ^defg^	1028.82 ± 53.33 ^efghijkl^	3.12 ± 0.52 ^ab^	14.24 ± 0.75 ^bcdefgh^	3.55 ± 0.25 ^abcde^	0.07 ± 0.02 ^c^	17.87 ± 0.34 ^defg^	117.55 ± 8.38 ^abc^
19	sandy loam	5.72 ± 0.44 ^cdefghi^	0.18 ± 0.02 ^cd^	4.03 ± 0.52 ^cdef^	0.24 ± 0.03 ^defgh^	1533.62 ± 141.61 ^efghijkl^	0.93 ± 0.03 ^cd^	6.35 ± 0.84 ^efghijkl^	1.52 ± 0.14 ^efghij^	0.01 ± 0.00 ^c^	17.40 ± 0.52 ^efg^	51.06 ± 7.05 ^efghij^
20	sandy loam	4.23 ± 0.13 ^jk^	0.09 ± 0.01 ^cd^	1.77 ± 0.11 ^ef^	0.12 ± 0.01 ^fgh^	785.34 ± 107.57 ^efghijkl^	0.35 ± 0.06 ^cd^	2.09 ± 0.35 ^l^	0.42 ± 0.14 ^j^	0.03 ± 0.02 ^c^	12.72 ± 0.25 ^fghi^	22.60 ± 3.77 ^ij^
21	sandy loam	6.69 ± 0.18 ^abcd^	0.17 ± 0.01 ^cd^	4.98 ± 0.26 ^cdef^	0.37 ± 0.03 ^defg^	1803.17 ± 115.79 ^defghi^	1.32 ± 0.21 ^cd^	14.48 ± 1.36 ^bcdefg^	2.00 ± 0.40 ^cdefghij^	0.09 ± 0.04 ^bc^	19.37 ± 1.06 ^def^	92.77 ± 11.04 ^abcdefgh^
22	loam	6.21 ± 0.09 ^abcdefg^	0.14 ± 0.02 ^cd^	3.10 ± 0.25 ^cdef^	0.16 ± 0.02 ^fgh^	613.78 ± 63.20 ^fghijkl^	0.41 ± 0.04 ^cd^	13.09 ± 0.31 ^bcdefghij^	2.24 ± 0.19 ^cdefghij^	0.10 ± 0.03 ^bc^	17.46 ± 1.07 ^efg^	91.23 ± 5.11 ^abcdefgh^
23	loam	6.41 ± 0.19 ^abcdef^	0.10 ± 0.01 ^cd^	2.18 ± 0.08 ^def^	0.13 ± 0.00 ^fgh^	289.91 ± 15.32 ^ijkl^	0.24 ± 0.03 ^cd^	7.54 ± 0.39 ^defghijkl^	0.51 ± 0.02 ^ij^	0.03 ± 0.00 ^c^	14.53 ± 0.42 ^efghi^	57.28 ± 2.08 ^cdefghij^
24	sandy loam	6.11 ± 0.06 ^bcdefgh^	0.06 ± 0.00 ^d^	1.20 ± 0.08 ^ef^	0.10 ± 0.00 ^gh^	345.59 ± 64.84 ^ijkl^	0.17 ± 0.02 ^d^	4.78 ± 0.18 ^hijkl^	0.95 ± 0.07 ^ghij^	0.01 ± 0.00 ^c^	12.42 ± 1.07 ^fghi^	48.05 ± 2.87 ^fghij^
25	sandy loam	5.94 ± 0.40 ^bcdefghi^	0.12 ± 0.04 ^cd^	4.30 ± 0.96 ^cdef^	0.21 ± 0.05 ^defgh^	229.94 ± 105.17 ^ijkl^	0.48 ± 0.15 ^cd^	9.74 ± 3.16 ^bcdefghijkl^	1.43 ± 0.47 ^efghij^	0.09 ± 0.03 ^bc^	16.49 ± 1.85 ^efg^	68.19 ± 13.81 ^bcdefghij^
26	sandy loam	6.22 ± 0.11 ^abcdefg^	0.18 ± 0.03 ^cd^	7.05 ± 0.97 ^bcd^	0.44 ± 0.04 ^bcde^	1946.34 ± 447.05 ^defgh^	0.89 ± 0.13 ^cd^	11.78 ± 2.25 ^bcdefghijk^	1.33 ± 0.46 ^efghij^	0.03 ± 0.01 ^c^	21.39 ± 2.54 ^cde^	64.54 ± 5.40 ^bcdefghij^
27	sandy loam	5.21 ± 0.13 ^fghijk^	0.14 ± 0.02 ^cd^	3.57 ± 0.42 ^cdef^	0.19 ± 0.03 ^defgh^	486.49 ± 74.72 ^ghijkl^	0.77 ± 0.27 ^cd^	5.56 ± 1.17 ^fghijkl^	0.97 ± 0.23 ^ghij^	0.02 ± 0.01 ^c^	15.32 ± 0.45 ^efghi^	48.55 ± 12.08 ^fghij^
28	sandy clay loam	4.86 ± 0.18 ^ijk^	0.13 ± 0.01 ^cd^	2.10 ± 0.31 ^def^	0.13 ± 0.00 ^fgh^	1471.30 ± 131.10 ^efghijkl^	0.89 ± 0.06 ^cd^	4.55 ± 0.78 ^ijkl^	1.00 ± 0.27 ^ghij^	0.01 ± 0.00 ^c^	14.46 ± 0.18 ^efghi^	44.58 ± 6.50 ^ghij^
29	sandy loam	6.30 ± 0.10 ^abcdefg^	0.53 ± 0.08 ^ab^	14.84 ± 2.92 ^a^	0.86 ± 0.16 ^a^	5344.04 ± 797.78 ^a^	1.95 ± 0.33 ^abc^	29.06 ± 4.27 ^a^	5.35 ± 0.91 ^ab^	0.06 ± 0.02 ^c^	29.53 ± 0.36 ^abc^	123.77 ± 19.40 ^ab^
30	sandy loam	5.33 ± 0.18 ^fghij^	0.15 ± 0.01 ^cd^	3.85 ± 0.20 ^cdef^	0.21 ± 0.03 ^defgh^	147.26 ± 8.26 ^kl^	0.24 ± 0.05 ^cd^	9.10 ± 0.63 ^bcdefghijkl^	1.14 ± 0.07 ^fghij^	0.04 ± 0.02 ^c^	17.36 ± 0.81 ^efg^	61.19 ± 5.88 ^cdefghij^
31	loam	4.81 ± 0.18 ^Ijk^	0.16 ± 0.01 ^cd^	4.63 ± 0.18 ^cdef^	0.30 ± 0.02 ^defgh^	1051.28 ± 163.55 ^efghijkl^	0.36 ± 0.01 ^cd^	3.22 ± 0.52 ^kl^	0.81 ± 0.14 ^ghij^	0.01 ± 0.00 ^c^	15.33 ± 1.05 ^efghi^	28.34 ± 2.61 ^ij^
32	sandy loam	6.62 ± 0.04 ^abcde^	0.23 ± 0.01 ^cd^	5.05 ± 0.05 ^cdef^	0.30 ± 0.01 ^defgh^	3219.11 ± 171.88 ^bcd^	1.58 ± 0.21 ^abcd^	18.25 ± 1.28 ^b^	3.24 ± 0.06 ^bcdef^	0.07 ± 0.01 ^c^	18.62 ± 0.29 ^defg^	124.46 ± 8.42 ^ab^
33	sandy loam	5.15 ± 0.13 ^ghijk^	0.07 ± 0.01 ^d^	0.84 ± 0.04 ^f^	0.08 ± 0.00 ^h^	231.56 ± 24.48 ^jkl^	0.29 ± 0.03 ^cd^	7.00 ± 0.93 ^defghijkl^	1.38 ± 0.11 ^efghij^	0.01 ± 0.00 ^c^	7.96 ± 0.98 ^hi^	109.71 ± 6.27 ^abcde^
34	sandy loam	7.11 ± 0.13 ^ab^	0.15 ± 0.02 ^cd^	2.43 ± 0.13 ^def^	0.14 ± 0.00 ^fgh^	1257.68 ± 35.89 ^efghijkl^	0.35 ± 0.01 ^cd^	9.45 ± 0.82 ^bcdefghijkl^	0.85 ± 0.09 ^ghij^	0.02 ± 0.00 ^c^	14.46 ± 0.09 ^efghi^	73.69 ± 6.36 ^abcdefghi^
35	sandy clay loam	4.08 ± 0.04 ^k^	0.11 ± 0.01 ^cd^	1.27 ± 0.14 ^ef^	0.11 ± 0.00 ^gh^	617.59 ± 92.98 ^fghijkl^	0.29 ± 0.06 ^cd^	0.57 ± 0.04 ^l^	0.17 ± 0.05 ^j^	0.02 ± 0.00 ^c^	10.22 ± 1.81 ^ghi^	11.16 ± 2.77 ^j^
36	sandy loam	6.22 ± 0.08 ^abcdefg^	0.07 ± 0.01 ^d^	0.75 ± 0.10 ^f^	0.07 ± 0.01 ^h^	197.18 ± 46.84 ^jkl^	0.25 ± 0.02 ^cd^	7.58 ± 0.50 ^defghijkl^	2.08 ± 0.43 ^cdefghij^	0.02 ± 0.01 ^c^	7.80 ± 1.19 ^i^	130.71 ± 14.65 ^a^

EC: electrical conductivity; OM: organic matter; TN: total nitrogen; AP: available phosphate; CEC: cation exchange capacity; BS: base saturation. Mean values represented with different letters are significantly different according to Tukey’s test (*p* < 0.05).

**Table 2:** Growth characteristics of *Schisandra chinensis* fruit in 36 different cultivation sites.

Cultivation Sites (n = 3)	NFB	LB	WB	FWB	LF	WF	FWF	FW30F	SG
		(mm)	(mm)	(g)	(mm)	(mm)	(g)	(g)	(Brix°)
1	25.89 ± 2.08 ^ab^	53.77 ± 2.88 ^abc^	21.76 ± 0.68 ^a^	13.22 ± 1.47 ^ab^	09.52 ± 0.01 ^a^	08.31 ± 0.20 ^a^	0.60 ± 0.02 ^abc^	19.86 ± 1.00 ^abcd^	07.79 ± 0.49 ^bcd^
2	21.11 ± 1.74 ^b^	51.42 ± 0.84 ^abc^	19.45 ± 1.15 ^a^	10.58 ± 0.37 ^ab^	09.54 ± 0.14 ^a^	08.75 ± 0.26 ^a^	0.63 ± 0.03 ^abc^	19.53 ± 0.58 ^abcd^	08.94 ± 0.31 ^abcd^
3	30.78 ± 2.63 ^ab^	64.36 ± 1.58 ^abc^	23.69 ± 1.25 ^a^	19.11 ± 2.10 ^ab^	11.28 ± 0.47 ^a^	09.66 ± 0.23 ^a^	0.71 ± 0.04 ^abc^	23.46 ± 0.52 ^abcd^	09.06 ± 0.63 ^abcd^
4	25.89 ± 0.59 ^ab^	81.41 ± 8.71 ^a^	24.29 ± 1.12 ^a^	19.65 ± 0.57 ^ab^	12.48 ± 0.09 ^a^	10.64 ± 0.19 ^a^	0.91 ± 0.02 ^a^	28.39 ± 0.51 ^abc^	10.44 ± 0.67 ^ab^
5	21.11 ± 0.78 ^b^	50.89 ± 4.08 ^bc^	19.25 ± 1.89 ^a^	11.32 ± 2.22 ^ab^	09.87 ± 0.93 ^a^	08.86 ± 0.75 ^a^	0.66 ± 0.15 ^abc^	19.40 ± 3.90 ^abcd^	09.79 ± 0.38 ^abc^
6	31.11 ± 4.81 ^ab^	65.07 ± 10.60 ^abc^	21.58 ± 1.12 ^a^	15.98 ± 2.40 ^ab^	10.07 ± 0.71 ^a^	08.78 ± 0.36 ^a^	0.57 ± 0.03 ^abc^	18.55 ± 0.40 ^abcd^	08.99 ± 0.29 ^abcd^
7	30.11 ± 0.48 ^ab^	74.24 ± 2.48 ^abc^	24.59 ± 1.28 ^a^	20.05 ± 1.68 ^ab^	10.87 ± 1.03 ^a^	09.78 ± 0.54 ^a^	0.74 ± 0.05 ^abc^	22.17 ± 1.53 ^abcd^	06.78 ± 0.33 ^bcd^
8	23.89 ± 2.45 ^ab^	58.60 ± 6.88 ^abc^	18.71 ± 1.19 ^a^	10.33 ± 1.77 ^ab^	09.41 ± 0.52 ^a^	08.75 ± 0.54 ^a^	0.51 ± 0.05 ^bc^	17.20 ± 0.68 ^cd^	04.74 ± 0.17 ^d^
9	26.00 ± 0.88 ^ab^	69.15 ± 1.66 ^abc^	23.65 ± 1.97 ^a^	17.59 ± 1.87 ^ab^	11.16 ± 1.17 ^a^	10.04 ± 0.90 ^a^	0.79 ± 0.12 ^abc^	26.55 ± 3.58 ^abcd^	05.89 ± 0.56 ^cd^
10	25.33 ± 1.53 ^ab^	53.62 ± 4.06 ^abc^	23.22 ± 0.39 ^a^	15.81 ± 1.65 ^ab^	10.16 ± 0.80 ^a^	08.68 ± 0.52 ^a^	0.74 ± 0.02 ^abc^	24.57 ± 2.46 ^abcd^	09.97 ± 0.74 ^abc^
11	29.89 ± 3.67 ^ab^	70.16 ± 4.85 ^abc^	22.51 ± 0.25 ^a^	20.25 ± 1.92 ^ab^	10.97 ± 0.72 ^a^	09.72 ± 0.54 ^a^	0.78 ± 0.06 ^abc^	25.03 ± 0.30 ^abcd^	09.78 ± 1.70 ^abc^
12	31.44 ± 2.16 ^ab^	69.26 ± 3.17 ^abc^	21.71 ± 0.34 ^a^	16.50 ± 1.80 ^ab^	10.51 ± 0.28 ^a^	09.32 ± 0.29 ^a^	0.64 ± 0.07 ^abc^	20.05 ± 2.48 ^abcd^	08.40 ± 1.44 ^abcd^
13	29.44 ± 1.61 ^ab^	69.50 ± 5.41 ^abc^	23.10 ± 0.63 ^a^	19.35 ± 0.39 ^ab^	10.29 ± 0.16 ^a^	09.25 ± 0.11 ^a^	0.76 ± 0.04 ^abc^	22.98 ± 1.07 ^abcd^	08.51 ± 0.39 ^abcd^
14	26.44 ± 2.13 ^ab^	70.04 ± 3.76 ^abc^	20.37 ± 0.10 ^a^	14.95 ± 1.68 ^ab^	10.97 ± 0.24 ^a^	09.89 ± 0.16 ^a^	0.70 ± 0.03 ^abc^	20.73 ± 0.70 ^abcd^	07.00 ± 0.76 ^bcd^
15	25.78 ± 1.64 ^ab^	69.74 ± 9.46 ^abc^	19.77 ± 1.81 ^a^	13.28 ± 0.98 ^ab^	10.81 ± 0.39 ^a^	09.44 ± 0.21 ^a^	0.63 ± 0.03 ^abc^	18.61 ± 0.70 ^abcd^	07.50 ± 1.25 ^bcd^
16	24.56 ± 1.54 ^ab^	60.67 ± 5.70 ^abc^	22.60 ± 2.02 ^a^	16.07 ± 2.59 ^ab^	11.20 ± 0.50 ^a^	09.89 ± 0.36 ^a^	0.76 ± 0.05 ^abc^	20.57 ± 0.96 ^abcd^	09.92 ± 1.71 ^abc^
17	31.89 ± 0.89 ^ab^	67.49 ± 0.78 ^abc^	23.63 ± 0.62 ^a^	19.16 ± 1.48 ^ab^	10.73 ± 0.18 ^a^	09.47 ± 0.09 ^a^	0.70 ± 0.02 ^abc^	22.13 ± 0.57 ^abcd^	08.96 ± 0.41 ^abcd^
18	30.56 ± 3.02 ^ab^	78.65 ± 5.05 ^ab^	22.31 ± 0.73 ^a^	18.60 ± 2.17 ^ab^	10.27 ± 0.28 ^a^	09.61 ± 0.29 ^a^	0.63 ± 0.07 ^abc^	17.69 ± 3.34 ^bcd^	10.26 ± 0.35 ^ab^
19	29.33 ± 1.58 ^ab^	73.15 ± 2.62 ^abc^	24.00 ± 1.61 ^a^	22.17 ± 3.13 ^a^	11.17 ± 0.68 ^a^	09.78 ± 0.54 ^a^	0.88 ± 0.13 ^ab^	26.33 ± 4.40 ^abcd^	08.16 ± 0.49 ^abcd^
20	22.89 ± 2.60 ^ab^	61.18 ± 7.32 ^abc^	19.52 ± 1.43 ^a^	14.96 ± 1.91 ^ab^	09.87 ± 0.18 ^a^	09.44 ± 0.69 ^a^	0.78 ± 0.04 ^abc^	22.02 ± 0.62 ^abcd^	08.70 ± 0.77 ^abcd^
21	28.44 ± 1.97 ^ab^	66.58 ± 2.94 ^abc^	21.83 ± 1.15 ^a^	16.38 ± 0.71 ^ab^	10.30 ± 0.45 ^a^	09.08 ± 0.23 ^a^	0.71 ± 0.02 ^abc^	22.28 ± 0.47 ^abcd^	09.71 ± 0.57 ^abc^
22	28.67 ± 2.27 ^ab^	61.72 ± 0.65 ^abc^	24.28 ± 0.61 ^a^	17.34 ± 1.77 ^a^	11.26 ± 0.41 ^a^	09.65 ± 0.28 ^a^	0.68 ± 0.05 ^abc^	24.24 ± 1.83 ^abcd^	08.01 ± 0.47 ^abcd^
23	26.56 ± 0.48 ^ab^	68.46 ± 9.49 ^abc^	22.78 ± 0.49 ^a^	17.65 ± 1.59 ^ab^	10.25 ± 0.57 ^a^	09.28 ± 0.38 ^a^	0.81 ± 0.06 ^abc^	23.45 ± 1.90 ^abcd^	08.33 ± 0.42 ^abcd^
24	32.22 ± 2.98 ^ab^	69.60 ± 1.56 ^abc^	23.22 ± 0.91 ^a^	18.73 ± 0.12 ^ab^	10.65 ± 0.67 ^a^	09.57 ± 0.53 ^a^	0.65 ± 0.06 ^abc^	20.46 ± 0.99 ^abcd^	10.01 ± 0.18 ^abc^
25	21.67 ± 1.95 ^ab^	44.48 ± 3.48 ^c^	19.60 ± 1.48 ^a^	08.62 ± 1.24 ^b^	09.52 ± 0.74 ^a^	08.27 ± 0.55 ^a^	0.49 ± 0.06 ^c^	15.39 ± 1.38 ^d^	08.76 ± 1.18 ^abcd^
26	28.11 ± 1.13 ^ab^	65.98 ± 1.48 ^abc^	23.45 ± 0.51 ^a^	21.07 ± 1.71 ^a^	10.44 ± 0.68 ^a^	09.30 ± 0.59 ^a^	0.92 ± 0.08 ^a^	28.77 ± 2.69 ^ab^	10.12 ± 1.13 ^abc^
27	27.56 ± 1.75 ^ab^	65.32 ± 10.79 ^abc^	22.77 ± 0.95 ^a^	18.18 ± 3.22 ^ab^	10.80 ± 0.76 ^a^	09.92 ± 0.83 ^a^	0.81 ± 0.06 ^abc^	25.47 ± 1.54 ^abcd^	07.47 ± 0.33 ^bcd^
28	27.00 ± 2.14 ^ab^	61.49 ± 5.26 ^abc^	22.23 ± 0.09 ^a^	16.86 ± 1.80 ^ab^	10.36 ± 0.78 ^a^	09.37 ± 0.56 ^a^	0.75 ± 0.07 ^abc^	23.54 ± 1.59 ^abcd^	09.56 ± 0.64 ^abc^
29	29.56 ± 1.39 ^ab^	67.22 ± 5.02 ^abc^	23.10 ± 1.60 ^a^	20.84 ± 3.77 ^a^	10.96 ± 0.94 ^a^	09.27 ± 0.56 ^a^	0.81 ± 0.09 ^abc^	24.84 ± 2.53 ^abcd^	09.19 ± 0.65 ^abc^
30	27.22 ± 3.13 ^ab^	70.29 ± 4.40 ^abc^	23.04 ± 2.23 ^a^	19.95 ± 4.24 ^ab^	11.07 ± 0.63 ^a^	09.75 ± 0.39 ^a^	0.90 ± 0.11 ^a^	29.81 ± 2.26 ^a^	08.04 ± 0.51 ^abcd^
31	29.78 ± 3.11 ^ab^	70.26 ± 5.15 ^abc^	24.57 ± 0.98 ^a^	17.09 ± 1.55 ^ab^	10.66 ± 0.10 ^a^	10.23 ± 0.37 ^a^	0.73 ± 0.03 ^abc^	23.54 ± 2.00 ^abcd^	09.26 ± 1.10 ^abc^
32	32.33 ± 1.95 ^ab^	64.87 ± 3.90 ^abc^	23.11 ± 1.45 ^a^	17.33 ± 1.88 ^ab^	10.39 ± 0.67 ^a^	09.34 ± 0.54 ^a^	0.61 ± 0.02 ^abc^	18.36 ± 0.48 ^bcd^	08.48 ± 0.24 ^abcd^
33	28.67 ± 0.84 ^ab^	68.20 ± 3.56 ^abc^	20.98 ± 1.96 ^a^	15.59 ± 2.27 ^ab^	10.29 ± 0.34 ^a^	09.00 ± 0.44 ^a^	0.63 ± 0.07 ^abc^	20.38 ± 2.02 ^abcd^	09.53 ± 0.44 ^abc^
34	33.56 ± 2.98 ^a^	76.07 ± 5.17 ^ab^	25.13 ± 1.39 ^a^	22.33 ± 2.58 ^a^	11.53 ± 0.85 ^a^	10.19 ± 0.63 ^a^	0.77 ± 0.04 ^abc^	24.79 ± 2.64 ^abcd^	06.51 ± 0.63 ^bcd^
35	31.67 ± 2.40 ^ab^	67.32 ± 5.77 ^abc^	23.12 ± 0.54 ^a^	20.46 ± 0.63 ^ab^	10.57 ± 0.21 ^a^	09.59 ± 0.37 ^a^	0.79 ± 0.02 ^abc^	24.02 ± 1.31 ^abcd^	12.23 ± 0.77 ^a^
36	25.89 ± 1.46 ^ab^	67.17 ± 5.70 ^abc^	24.16 ± 2.08 ^a^	19.79 ± 4.54 ^ab^	11.16 ± 0.55 ^a^	10.04 ± 0.66 ^a^	0.83 ± 0.15 ^abc^	25.67 ± 3.70 ^abcd^	08.78 ± 0.40 ^abcd^

NFB: number of fruit bunch; LB: length of fruit bunch; WB: width of fruit bunch; FWB: fresh weight of fruit bunch; LF: length of fruit; WF: width of fruit; FWF: fresh weight of fruit; FW30F: fresh weight of 30 fruits; SG: sugar contents. Mean values represented with different letters are significantly different according to Tukey’s test (*p* < 0.05).

**Table 3:** Linear regression, LOD, and LOQ of three marker compounds.

Compound	Regression Equation	Correlation Coefficient (*r*^2^)	Range (µg/mL)	LOD (µg/mL)	LOQ (µg/mL)
Schisandrin	Y = 7223X + 10,371	0.9999	6.25–400	0.003	0.01
Gomisin A	Y = 2743X − 322.44	1	6.25–400	0.02	0.07
Gomisin N	Y = 5990.5X − 4273.7	0.9999	6.25–400	0.01	0.04

LOD: limit of detection; LOQ: limit of quantification.

**Table 4:** Intra- and inter-day precision of three marker compounds.

Compound	Concentration (µg/mL)	Intra-Day ^a^ (n = 3)		Inter-Day ^b^ (n = 3)	
		Concentration Found (µg/mL)	RSD (%)	Concentration Found (µg/mL)	RSD (%)
Schisandrin	25	24.6	0.02	22.7	2.50
	100	103.5	0.52	101.8	1.70
	400	405.2	0.14	410.5	0.62
Gomisin A	25	25.9	0.05	26.2	1.54
	100	98.6	0.08	101.3	2.34
	400	399.4	0.29	409.5	1.98
Gomisin N	25	24.8	0.02	23.4	0.68
	100	102.7	0.02	96.8	0.23
	400	401.5	0.07	397.1	0.85

^a^ Sample analyzed three times on 1 day, n = 3; ^b^ Sample analyzed each day for three consecutive days, n = 3; RSD, relative standard deviation.

**Table 5:** Recoveries of three marker compounds.

Compound	Concentration (µg/mL)	Recovery (%) (n = 3)	RSD (%)
Schisandrin	25	98.40	2.81
	100	100.18	1.37
	400	100.81	0.38
Gomisin A	25	101.14	1.41
	100	100.48	1.40
	400	100.42	1.47
Gomisin N	25	100.82	1.88
	100	102.47	0.17
	400	102.68	1.07

RSD: relative standard deviation.

**Table 6:** Marker compounds composition of *Schisandra chinensis* in 36 different cultivation sites.

Cultivation Sites (n = 3)	Schisandrin (%)	Gomisin A (%)	Gomisin N (%)	Total (%)
1	0.52 ± 0.03 ^cdefg^	0.25 ± 0.06 ^abcdefg^	0.41 ± 0.02 ^abcdefg^	1.18 ± 0.04 ^hijk^
2	0.55 ± 0.02 ^cdefg^	0.41 ± 0.05 ^abcd^	0.45 ± 0.01 ^abcdef^	1.41 ± 0.02 ^cdef^
3	0.46 ± 0.02 ^defg^	0.41 ± 0.01 ^abcde^	0.29 ± 0.00 ^fg^	1.15 ± 0.02 ^hijk^
4	0.45 ± 0.01 ^defg^	0.17 ± 0.01 ^efg^	0.23 ± 0.01 ^g^	0.85 ± 0.02 ^n^
5	0.56 ± 0.02 ^bcdefg^	0.29 ± 0.02 ^abcdefg^	0.37 ± 0.05 ^abcdefg^	1.21 ± 0.04 ^ghij^
6	0.68 ± 0.06 ^abc^	0.32 ± 0.04 ^abcdefg^	0.54 ± 0.04 ^ab^	1.54 ± 0.02 ^bc^
7	0.61 ± 0.03 ^abcdefg^	0.29 ± 0.00 ^abcdefg^	0.55 ± 0.02 ^a^	1.46 ± 0.03 ^cde^
8	0.77 ± 0.00 ^ab^	0.49 ± 0.02 ^a^	0.49 ± 0.01 ^abcd^	1.75 ± 0.01 ^a^
9	0.46 ± 0.03 ^cdefg^	0.36 ± 0.03 ^abcdef^	0.33 ± 0.02 ^cdefg^	1.15 ± 0.02 ^hijk^
10	0.45 ± 0.03 ^efg^	0.16 ± 0.00 ^fg^	0.31 ± 0.03 ^efg^	0.91 ± 0.01 ^mn^
11	0.52 ± 0.01 ^cdefg^	0.20 ± 0.03 ^cdefg^	0.33 ± 0.01 ^cdefg^	1.05 ± 0.04 ^jklm^
12	0.47 ± 0.03 ^cdefg^	0.24 ± 0.03 ^bcdefg^	0.31 ± 0.02 ^defg^	1.02 ± 0.02 ^klm^
13	0.63 ± 0.06 ^abcdefg^	0.25 ± 0.02 ^abcdefg^	0.41 ± 0.05 ^abcdefg^	1.30 ± 0.03 ^efgh^
14	0.79 ± 0.03 ^a^	0.37 ± 0.06 ^abcdef^	0.46 ± 0.02 ^abcdef^	1.63 ± 0.06 ^ab^
15	0.62 ± 0.07 ^abcdefg^	0.44 ± 0.11 ^abcd^	0.46 ± 0.04 ^abcdef^	1.53 ± 0.05 ^bc^
16	0.59 ± 0.03 ^abcdefg^	0.31 ± 0.06 ^abcdefg^	0.40 ± 0.09 ^abcdefg^	1.30 ± 0.03 ^defgh^
17	0.67 ± 0.05 ^abcd^	0.37 ± 0.02 ^abcdef^	0.43 ± 0.03 ^abcdef^	1.47 ± 0.03 ^bcd^
18	0.45 ± 0.05 ^defg^	0.09 ± 0.04 ^g^	0.39 ± 0.06 ^abcdefg^	0.93 ± 0.05 ^mn^
19	0.67 ± 0.03 ^abcde^	0.22 ± 0.03 ^cdefg^	0.48 ± 0.01 ^abcde^	1.37 ± 0.02 ^cdefg^
20	0.48 ± 0.09 ^cdefg^	0.28 ± 0.08 ^abcdefg^	0.45 ± 0.03 ^abcdef^	1.21 ± 0.04 ^ghij^
21	0.41 ± 0.00 ^g^	0.27 ± 0.03 ^abcdefg^	0.35 ± 0.02 ^cdefg^	1.02 ± 0.01 ^klm^
22	0.47 ± 0.03 ^cdefg^	0.36 ± 0.05 ^abcdef^	0.35 ± 0.01 ^cdefg^	1.18 ± 0.03 ^hijk^
23	0.41 ± 0.03 ^fg^	0.23 ± 0.01 ^cdefg^	0.30 ± 0.02 ^fg^	0.94 ± 0.02 ^lmn^
24	0.42 ± 0.01 ^fg^	0.08 ± 0.01 ^g^	0.40 ± 0.01 ^abcdefg^	0.90 ± 0.01 ^mn^
25	0.44 ± 0.04 ^fg^	0.44 ± 0.09 ^abc^	0.28 ± 0.03 ^fg^	1.16 ± 0.04 ^hijk^
26	0.52 ± 0.02 ^cdefg^	0.27 ± 0.01 ^abcdefg^	0.36 ± 0.04 ^bcdefg^	1.15 ± 0.02 ^hijk^
27	0.42 ± 0.06 ^fg^	0.30 ± 0.06 ^abcdefg^	0.42 ± 0.05 ^abcdef^	1.14 ± 0.02 ^hijk^
28	0.53 ± 0.04 ^cdefg^	0.48 ± 0.04 ^ab^	0.28 ± 0.03 ^fg^	1.29 ± 0.04 ^efgh^
29	0.46 ± 0.06 ^cdefg^	0.32 ± 0.08 ^abcdefg^	0.39 ± 0.02 ^abcdefg^	1.17 ± 0.01 ^hijk^
30	0.51 ± 0.01 ^cdefg^	0.29 ± 0.00 ^abcdefg^	0.31 ± 0.02 ^defg^	1.11 ± 0.03 ^ijkl^
31	0.49 ± 0.03 ^cdefg^	0.27 ± 0.01 ^abcdefg^	0.39 ± 0.03 ^abcdefg^	1.15 ± 0.01 ^hijk^
32	0.55 ± 0.04 ^cdefg^	0.28 ± 0.01 ^abcdefg^	0.42 ± 0.01 ^abcdef^	1.26 ± 0.04 ^fghi^
33	0.59 ± 0.03 ^abcdefg^	0.20 ± 0.03 ^defg^	0.49 ± 0.03 ^abcde^	1.28 ± 0.03 ^fgh^
34	0.62 ± 0.05 ^abcdefg^	0.26 ± 0.01 ^abcdefg^	0.50 ± 0.03 ^abc^	1.39 ± 0.02 ^cdef^
35	0.47 ± 0.03 ^cdefg^	0.25 ± 0.04 ^abcdefg^	0.34 ± 0.01 ^cdefg^	1.05 ± 0.04 ^jklm^
36	0.51 ± 0.03 ^cdefg^	0.31 ± 0.02 ^abcdefg^	0.39 ± 0.06 ^abcdefg^	1.21 ± 0.02 ^ghij^

Mean values represented with different letters are significantly different according to Tukey’s test (*p* < 0.05).

## Data Availability

The data presented in this study are available under permission from the corresponding author upon reasonable request.

## Supplementary Materials

The following supporting information can be downloaded at: https://www.mdpi.com/article/10.3390/plants12223877/s1, Table S1: Meteorological data of 36 different *Schisandra chinensis* cultivation sites; Table S2: Pearson’s correlation coefficient between growth characteristics and soil physicochemical properties of *Schisandra chinensis*; Table S3: Pearson’s correlation coefficient between growth characteristics and meteorological properties of *Schisandra chinensis*; Table S4: Pearson’s correlation coefficient between growth characteristics and marker compounds of *Schisandra chinensis*; Table S5: Pearson’s correlation coefficient between soil physicochemical properties of *Schisandra chinensis* fruit cultivation sites; Table S6: Pearson’s correlation coefficient between growth characteristics of *Schisandra chinensis* fruit; Table S7: Geographic information about the cultivation sites where fruits of *Schisandra chinensis* were collected in South Korea.
