# Supplementary material for: Multivariate Analysis among Marker Compounds, Environmental Factors, and Fruit Quality of Schisandra chinensis at Different Locations in South Korea
Source: Plants (Basel). 2023 Nov 16;12(22):3877. doi: 10.3390/plants12223877 (PMC10675420; doi:10.3390/plants12223877)
Supplement: Supplementary file 1 [file plants-12-03877-s001.zip › plants-2698403-supplementary.pdf]

SUPPLEMENTARY DATA

Multivariate Analysis among Marker Compounds, Environmental Factors, and Fruit Quality of *Schisandra chinensis* at Different Locations in South Korea

Dong Hwan Lee [1], Young-Ki Kim [1], Yonghwan Son [1], Gwang Hun Park [1], Hae-Yun Kwon [1], Youngki Park [2], Eung-Jun Park [2], Sun-Young Lee [1] and Hyun-Jun Kim [1,*]

1 Forest Medicinal Resources Research Center, National Institute of Forest Science, Yeongju-si 36040, Republic of Korea; leedh0419@korea.kr (D.H.L.); treeace@korea.kr (Y.-K.K.); thsdydghks@korea.kr (Y.S.); ppkh0230@korea.kr (G.H.P.); kwonhy05@korea.kr (H.-Y. K.); nararawood@korea.kr (S.-Y.L.)

2 Department of Forest Bioresources, National Institute of Forest Science, Suwon 16631, Republic of Korea; woodpark@korea.kr (Y.P.); pahkej@korea.kr (E.-J.P.)

* Correspondence: mind4938@korea.kr

Table S1. Meteorological data of 36 different *Schisandra chinensis* cultivation sites

Cultivation Sites	AAT (℃)	AAMT (℃)	AAmT (℃)	AMT (℃)	AmT (℃)	TP (mm)	SSH (h)
1	13.3	19.3	8.3	36.2	-9.5	2136.8	2161.2
2	12.5	18.0	7.5	34.0	-13.3	1694.4	2184.3
3	12.5	18.0	7.5	34.0	-13.3	1694.4	2184.3
4	12.5	18.0	7.5	34.0	-13.3	1694.4	2184.3
5	11.0	17.1	5.3	33.9	-20.3	1551.8	2152.3
6	11.2	17.3	6.2	35.1	-16.7	1429.1	1901.8
7	11.2	17.3	6.0	33.2	-13.1	2126.0	1988.7
8	12.5	18.0	7.5	34.0	-13.3	1694.4	2184.3
9	12.5	18.0	7.5	34.0	-13.3	1694.4	2184.3
10	13.9	19.9	8.7	36.9	-10.5	1371.6	2368.8
11	11.2	17.3	6.0	33.2	-13.1	2126.0	1988.7
12	11.2	17.3	6.0	33.2	-13.1	2126.0	1988.7
13	12.6	18.0	8.0	34.9	-10.5	1488.7	2385.3
14	12.2	18.0	7.1	36.4	-17.2	1550.9	2284.5
15	12.5	18.0	7.5	34.0	-13.3	1694.4	2184.3
16	12.1	18.1	6.8	34.5	-17.0	1446.5	2222.0
17	12.8	17.9	8.2	34.5	-14.6	1317.0	2279.5
18	11.5	18.5	5.7	36.4	-16.3	1285.8	2190.8
19	11.2	17.3	6.0	33.2	-13.1	2126.0	1988.7
20	12.2	18.0	7.0	34.1	-16.0	1443.5	2185.3
21	11.2	17.3	6.2	35.1	-16.7	1429.1	1901.8
22	13.1	19.1	8.1	34.7	-12.3	2007.2	2162.1
23	12.5	18.7	7.2	34.4	-15.4	1758.2	2062.0
24	9.6	15.0	4.9	34.9	-17.1	1530.1	2022.1
25	11.2	17.3	6.0	33.2	-13.1	2126.0	1988.7
26	13.0	19.7	7.5	36.3	-10.4	1910.6	2176.8
27	12.8	18.0	8.0	34.7	-12.6	1609.7	2356.9
28	13.9	18.9	9.7	36.0	-13.7	1358.6	2319.9
29	13.9	19.6	9.0	35.1	-10.1	1469.5	2254.7
30	10.8	16.9	5.3	32.8	-17.7	1297.3	2076.4
31	13.1	18.8	8.2	35.0	-13.0	1563.7	2271.9
32	12.0	17.6	6.7	33.5	-14.3	1499.9	2211.4
33	12.9	18.0	8.5	34.9	-12.6	1635.5	2444.2
34	12.5	18.0	7.5	34.0	-13.3	1694.4	2184.3
35	12.3	19.5	6.1	35.9	-15.9	1128.9	2174.3
36	11.0	17.4	6.0	36.0	-17.8	1305.4	1928.5

Table S2. Pearson's correlation coefficient between growth characteristics and soil physico-chemical properties of *Schisandra chinensis*

	Correlation coefficient (r) [a]								
	NFB	LB	WB	FWB	LF	WF	FWF	FW30F	SG
pH	-0.213*	-0.148	-0.093	-0.208*	0.036	0.045	-0.098	-0.097	-0.046
	(0.027)	(0.125)	(0.338)	(0.031)	(0.713)	(0.642)	(0.311)	(0.318)	(0.638)
EC	0.074	0.011	-0.087	-0.006	-0.069	-0.118	-0.110	-0.090	-0.241*
	(0.449)	(0.911)	(0.369)	(0.955)	(0.479)	(0.223)	(0.258)	(0.355)	(0.012)
OM	0.287**	0.120	0.137	0.100	0.107	0.017	-0.141	-0.167	-0.067
	(0.003)	(0.217)	(0.157)	(0.302)	(0.051)	(0.860)	(0.147)	(0.083)	(0.488)
TN	-0.032	-0.011	-0.021	0.001	0.051	0.029	0.053	-0.065	0.004
	(0.741)	(0.910)	(0.831)	(0.995)	(0.600)	(0.764)	(0.588)	(0.504)	(0.964)
AP	0.033	0.003	-0.054	0.019	-0.088	-0.136	-0.050	-0.050	-0.121
	(0.737)	(0.973)	(0.577)	(0.842)	(0.364)	(0.161)	(0.610)	(0.607)	(0.213)
K^+	0.073	0.032	-0.048	0.039	-0.079	-0.125	-0.043	-0.060	-0.083
	(0.453)	(0.741)	(0.623)	(0.689)	(0.418)	(0.199)	(0.659)	(0.537)	(0.390)
Ca^{2+}	0.183	0.077	-0.002	0.110	0.016	-0.070	-0.008	-0.045	0.129
	(0.058)	(0.429)	(0.980)	(0.257)	(0.867)	(0.471)	(0.937)	(0.647)	(0.184)
Mg^{2+}	0.063	-0.013	-0.155	-0.084	-0.093	-0.098	-0.197*	-0.258**	0.166
	(0.516)	(0.892)	(0.108)	(0.390)	(0.340)	(0.312)	(0.041)	(0.007)	(0.085)
Na^+	0.146	0.005	-0.029	0.025	-0.030	-0.120	-0.127	-0.147	-0.071
	(0.130)	(0.955)	(0.769)	(0.795)	(0.756)	(0.215)	(0.189)	(0.130)	(0.464)
CEC	0.226*	0.017	-0.023	0.025	-0.003	-0.104	-0.194*	-0.202*	0.091
	(0.019)	(0.864)	(0.815)	(0.799)	(0.979)	(0.285)	(0.045)	(0.036)	(0.348)
BS	0.083	-0.034	0.002	-0.038	-0.005	-0.072	-0.155	-0.187	0.075
	(0.393)	(0.726)	(0.987)	(0.696)	(0.958)	(0.460)	(0.108)	(0.053)	(0.440)
Sand	0.210*	0.048	-0.107	0.017	-0.045	-0.059	-0.147	-0.158	0.029
	(0.029)	(0.622)	(0.272)	(0.861)	(0.640)	(0.542)	(0.128)	(0.102)	(0.765)
Silt	-0.188	0.038	0.032	-0.041	0.068	0.109	0.078	0.048	-0.137
	(0.051)	(0.693)	(0.743)	(0.673)	(0.482)	(0.259)	(0.425)	(0.621)	(0.156)
Clay	-0.086	-0.140	0.138	0.031	-0.022	-0.059	0.141	0.203*	0.153
	(0.376)	(0.149)	(0.155)	(0.749)	(0.819)	(0.544)	(0.147)	(0.035)	(0.115)

[a] Correlation coefficient (r) written is significantly correlated between the variables compared. Positive values denote positive correlation and negative values denote negative correlation. Values in bracket means p value (** $p < 0.01$, * $p < 0.05$)

Table S3. Pearson's correlation coefficient between growth characteristics and meteorological properties of *Schisandra chinensis*

	Correlation coefficient (r) [a]								
	NFB	LB	WB	FWB	LF	WF	FWF	FW30F	SG
AAT	-0.070	-0.110	0.064	-0.017	-0.013	-0.048	0.082	0.144	-0.040
	(0.472)	(0.256)	(0.514)	(0.860)	(0.896)	(0.619)	(0.399)	(0.136)	(0.683)
AAMT	-0.060	-0.100	0.090	0.043	-0.043	-0.068	0.143	0.169	0.116
	(0.539)	(0.304)	(0.353)	(0.661)	(0.661)	(0.485)	(0.141)	(0.080)	(0.233)
AAmT	-0.044	-0.091	0.066	-0.027	0.000	-0.043	0.039	0.120	-0.104
	(0.652)	(0.348)	(0.499)	(0.784)	(0.999)	(0.656)	(0.688)	(0.215)	(0.282)
AMT	0.026	-0.041	0.052	0.039	-0.094	-0.077	0.033	0.025	0.272**
	(0.793)	(0.673)	(0.594)	(0.691)	(0.333)	(0.428)	(0.738)	(0.799)	(0.004)
AmT	0.086	-0.037	0.152	0.075	-0.019	-0.110	0.027	0.131	-0.138
	(0.376)	(0.701)	(0.117)	(0.438)	(0.848)	(0.255)	(0.780)	(0.176)	(0.153)
TP	-0.052	-0.069	-0.009	-0.088	-0.008	-0.085	-0.098	-0.044	-0.283**
	(0.594)	(0.481)	(0.923)	(0.367)	(0.934)	(0.384)	(0.310)	(0.653)	(0.003)
SSH	-0.048	-0.039	-0.014	-0.035	-0.006	0.026	0.034	0.045	0.012
	(0.622)	(0.690)	(0.885)	(0.718)	(0.950)	(0.793)	(0.724)	(0.645)	(0.904)
ALT	0.132	0.119	0.216*	0.175	0.176	0.112	0.060	0.131	0.177
	(0.173)	(0.221)	(0.025)	(0.071)	(0.069)	(0.247)	(0.538)	(0.177)	(0.068)

[a] Correlation coefficient (r) written is significantly correlated between the variables compared. Positive values denote positive correlation and negative values denote negative correlation. Values in bracket means p value (** $p < 0.01$, * $p < 0.05$)

Table S4. Pearson's correlation coefficient between growth characteristics and marker compounds of *Schisandra chinensis*

	Correlation coefficient (r) [a]								
	NFB	LB	WB	FWB	LF	WF	FWF	FW30F	SG
Schisandrin	0.056	0.060	-0.157	-0.066	-0.072	-0.050	-0.173	-0.157	-0.307**
	(0.567)	(0.537)	(0.104)	(0.496)	(0.457)	(0.605)	(0.074)	(0.104)	(0.001)
Gomisin A	-0.257**	-0.266**	-0.193*	-0.254**	-0.076	-0.081	-0.113	-0.069	-0.293**
	(0.007)	(0.005)	(0.045)	(0.008)	(0.434)	(0.407)	(0.246)	(0.476)	(0.002)
Gomisin N	0.105	0.099	-0.074	-0.067	-0.039	0.028	-0.205*	-0.291**	-0.308**
	(0.280)	(0.307)	(0.449)	(0.489)	(0.690)	(0.770)	(0.033)	(0.002)	(0.001)
Total	-0.062	-0.067	-0.218*	-0.199*	-0.095	-0.057	-0.239*	-0.245*	-0.450**
	(0.523)	(0.491)	(0.024)	(0.039)	(0.326)	(0.555)	(0.013)	(0.011)	(0.000)

[a] Correlation coefficient (r) written is significantly correlated between the variables compared. Positive values denote positive correlation and negative values denote negative correlation. Values in bracket means p value (** $p < 0.01$, * $p < 0.05$).

Table S5. Pearson's correlation coefficient between soil physicochemical properties of *Schisandra chinensis* fruit cultivation sites

	Correlation coefficient (*r*) [a]													
	pH	EC	OM	TN	AP	K	Ca	Mg	Na	CEC	BS	Sand	Silt	Clay
pH	-	0.114 (0.239)	0.184 (0.057)	0.211* (0.028)	0.257** (0.007)	0.367** (0.000)	0.585** (0.000)	0.588** (0.000)	0.077 (0.425)	0.187 (0.052)	0.684** (0.000)	0.234* (0.015)	-0.063 (0.516)	-0.311** (0.001)
EC		-	0.624** (0.000)	0.650** (0.000)	0.637** (0.000)	0.672** (0.000)	0.596** (0.000)	0.508** (0.000)	0.488** (0.000)	0.542** (0.000)	0.336** (0.000)	0.010 (0.921)	0.063 (0.514)	-0.110 (0.255)
OM			-	0.981** (0.000)	0.737** (0.000)	0.457** (0.000)	0.744** (0.000)	0.593** (0.000)	0.169 (0.080)	0.855** (0.000)	0.216* (0.025)	0.074 (0.449)	0.011 (0.913)	-0.143 (0.140)
TN				-	0.758** (0.000)	0.496** (0.000)	0.770** (0.000)	0.617** (0.000)	0.156 (0.107)	0.862** (0.000)	0.250** (0.009)	0.094 (0.331)	0.009 (0.922)	-0.178 (0.066)
AP					-	0.609** (0.000)	0.725** (0.000)	0.677** (0.000)	0.181 (0.061)	0.607** (0.000)	0.364** (0.000)	0.157 (0.104)	-0.047 (0.628)	-0.203* (0.035)
K						-	0.592** (0.000)	0.667** (0.000)	0.503** (0.000)	0.413** (0.000)	0.497** (0.000)	0.071 (0.462)	0.061 (0.531)	-0.214* (0.026)
Ca							-	0.819** (0.000)	0.209* (0.030)	0.679** (0.000)	0.699** (0.000)	0.209* (0.030)	-0.078 (0.423)	-0.247** (0.010)
Mg								-	0.218* (0.023)	0.491** (0.000)	0.701** (0.000)	0.112 (0.248)	-0.041 (0.676)	-0.134 (0.167)
Na									-	0.140 (0.150)	0.276** (0.004)	-0.022 (0.820)	0.077 (0.431)	-0.075 (0.442)
CEC										-	0.047 (0.629)	-0.022 (0.818)	0.131 (0.175)	-0.155 (0.108)
BS											-	0.331** (0.000)	-0.202* (0.036)	-0.275** (0.004)
Sand												-	-.817** (0.000)	-0.525** (0.000)
Silt													-	-0.061 (0.528)
Clay														-

[a] Correlation coefficient (*r*) written is significantly correlated between the variables compared. Positive values denote positive correlation and negative values denote negative correlation. Values in bracket means *p* value (** $p < 0.01$, * $p < 0.05$).

Table S6. Pearson's correlation coefficient between growth characteristics of *Schisandra chinensis* fruit

	Correlation coefficient (*r*) [a]								
	NFB	LB	WB	FWB	LF	WF	FWF	FW30F	SG
NFB	-	0.651** (0.000)	0.472** (0.000)	0.672** (0.000)	0.243* (0.011)	0.222* (0.021)	0.088 (0.366)	0.113 (0.243)	-0.021 (0.827)
LB		-	0.413** (0.000)	0.696** (0.000)	0.437** (0.000)	0.513** (0.000)	0.384** (0.000)	0.357** (0.000)	-0.064 (0.508)
WB			-	0.732** (0.000)	0.689** (0.000)	0.648** (0.000)	0.584** (0.000)	0.549** (0.000)	-0.013 (0.897)
FWB				-	0.557** (0.000)	0.543** (0.000)	0.744** (0.000)	0.689** (0.000)	0.059 (0.542)
LF					-	0.887** (0.000)	0.591** (0.000)	0.509** (0.000)	-0.029 (0.766)
WF						-	0.610** (0.000)	0.532** (0.000)	-0.100 (0.303)
FWF							-	0.886** (0.000)	0.135 (0.163)
FW30F								-	0.066 (0.498)
SG									-

[a] Correlation coefficient (*r*) written is significantly correlated between the variables compared. Positive values denote positive correlation and negative values denote negative correlation. Values in bracket means *p* value (** $p < 0.01$, * $p < 0.05$).

Table S7. Geographic information about the cultivation sites where fruits of *Schisandra chinensis* were collected in South Korea

Cultivation sites	name of sites	Altitude (m)	N (latitude)	E (longitude)
1	Wonju-si	360	37.207500	128.080000
2	Inje-gun	250	37.876111	128.281389
3	Taebaek-si	470	37.261389	128.995000
4	Pyeongchang-gun	566	37.412222	128.480556
5	Gapyeong-gun	381	37.814444	127.483689
6	Gwangju-si	370	37.398056	127.320556
7	Geochang-gun	452	35.781111	128.008889
8	Sancheong-gun	112	35.410556	127.789722
9	Hamyang-gun	146	35.539722	127.613611
10	Gyeongju-si	170	35.758056	128.981389
11	Gimcheon-si	474	35.997222	127.926111
12	Mungyeong-si 1	327	36.591389	128.011667
13	Mungyeong-si 2	80	36.685833	128.253333
14	Mungyeong-si 3	280	36.797500	128.219444
15	Mungyeong-si 4	165	36.691389	128.253333
16	Mungyeong-si 5	99	36.766667	128.364167
17	Mungyeong-si 6	340	36.565472	128.263583
18	Mungyeong-si 7	500	36.641111	128.217778
19	Mungyeong-si 8	506	36.629444	128.058333
20	Bonghwa-gun	77	36.828056	129.000278
21	Sangju-si	360	36.590278	127.924167
22	Yeongyang-gun	552	36.714167	129.102778
23	Yeongju-si	82	36.940833	128.653056
24	Uiseong-gun	756	36.496389	128.463333
25	Namwon-si	486	35.403889	127.529333
26	Muju-gun	460	35.853611	127.681667
27	Sunchang-gun	183	35.447194	126.915611
28	Jangsu-gun 1	299	35.691139	127.490778
29	Jangsu-gun 2	310	35.686389	127.621972
30	Gongju-si	503	36.636667	126.982778
31	Nonsan-si	394	36.112778	127.233333
32	Seosan-si	226	36.835278	126.409722
33	Cheonan-si	112	36.749167	127.160000
34	Boeun-gun	171	36.433333	127.783333
35	Cheongju-si	93	36.603611	127.633056
36	Chungju-si	591	36.990000	127.808889
